# The post-translational modification O-GlcNAc is a sensor and regulator of metabolism

**DOI:** 10.1098/rsob.240209

**Published:** 2024-10-30

**Authors:** Murielle M. Morales, Matthew R. Pratt

**Affiliations:** ^1^ Department of Biological Sciences, University of Southern California, Los Angeles, CA 90089, USA; ^2^ Department of Chemistry, University of Southern California, Los Angeles, CA 90089, USA

**Keywords:** post-translational, modifications, O-GlcNAc, sensor, regulators, metabolism

## Abstract

Cells must rapidly adapt to changes in nutrient conditions through responsive signalling cascades to maintain homeostasis. One of these adaptive pathways results in the post-translational modification of proteins by O-GlcNAc. O-GlcNAc modifies thousands of nuclear and cytoplasmic proteins in response to nutrient availability through the hexosamine biosynthetic pathway. O-GlcNAc is highly dynamic and can be added and removed from proteins multiple times throughout their life cycle, setting it up to be an ideal regulator of cellular processes in response to metabolic changes. Here, we describe the link between cellular metabolism and O-GlcNAc, and we explore O-GlcNAc’s role in regulating cellular processes in response to nutrient levels. Specifically, we discuss the mechanisms of elevated O-GlcNAc levels in contributing to diabetes and cancer, as well as the role of decreased O-GlcNAc levels in neurodegeneration. These studies form a foundational understanding of aberrant O-GlcNAc in human disease and provide an opportunity to further improve disease identification and treatment.

## 1. Introduction

Cellular metabolism is encompassed by the sum of chemical reactions necessary to sustain homeostasis. This process is mediated by multiple enzymes in key pathways that regulate the production of energy, through the generation of ATP, to run cellular processes like the synthesis of building blocks for cellular structures and macromolecules, and the elimination of metabolic waste. Importantly, cellular metabolism relies on nutrient sensing mechanisms to respond to changes in the environment. One of these mechanisms is the post-translational modification O-GlcNAc. O-GlcNAc modifies thousands of intracellular proteins through the attachment of an O-linked N-acetylglucosamine moiety onto serine and threonine residues. Unlike other forms of glycosylation, O-GlcNAc is a single monosaccharide that is not further elaborated into complex oligosaccharides. Furthermore, O-GlcNAc is dynamically added and removed from proteins in response to changes in the cellular environment. This rapid cycling on substrates enables O-GlcNAc to act as important regulator of proteins. The addition of O-GlcNAc onto proteins has been demonstrated to have diverse effects and can alter protein activity, interactions, stability and subcellular localization.

The importance of O-GlcNAc in regulating signalling pathways is highlighted by its crosstalk with protein phosphorylation. O-GlcNAc and phosphorylation participate in various forms of interplay, where the presence of one modification can influence the presence of the other. Both modifications are attached onto serine or threonine residues and several studies have investigated their direct competition to modify the same sites [1]. They can also modify residues in close proximity, causing steric hindrance towards the addition of the other modification. However, unlike the hundreds of unique kinases and phosphatases that regulate phosphorylation, O-GlcNAc is only added and removed by two enzymes. O-GlcNAc transferase (OGT) uses the high energy donor sugar UDP-GlcNAc to add GlcNAc onto protein substrates and O-GlcNAcase (OGA) removes it.

OGT and OGA are highly conserved across species, demonstrating that O-GlcNAc is essential for cellular function. In mice, it was found that OGT is located on the X chromosome and gene deletion of OGT is embryonically lethal [2]. Similarly, deletion of OGA in mice resulted in nearly complete perinatal lethality [3]. Changes in the overall levels of O-GlcNAc are associated with several human diseases. For example, O-GlcNAc levels are increased in both diabetes and cancer, where cells undergo disruptions in energy metabolism and the stress response [4–7]. By contrast, O-GlcNAc levels are lowered in neurodegeneration, with Alzheimer’s disease-affected brain tissue displaying decreased O-GlcNAc levels compared with age-matched controls [8,9]. In this review, we further explore the role of O-GlcNAc in cellular metabolism and we provide an overview of the broad contours of O-GlcNAc and metabolism in human disease. We direct interested reviewers to these more comprehensive reviews [10–15].

## 2. O-GlcNAc is a nutrient sensor

**Figure 1.**
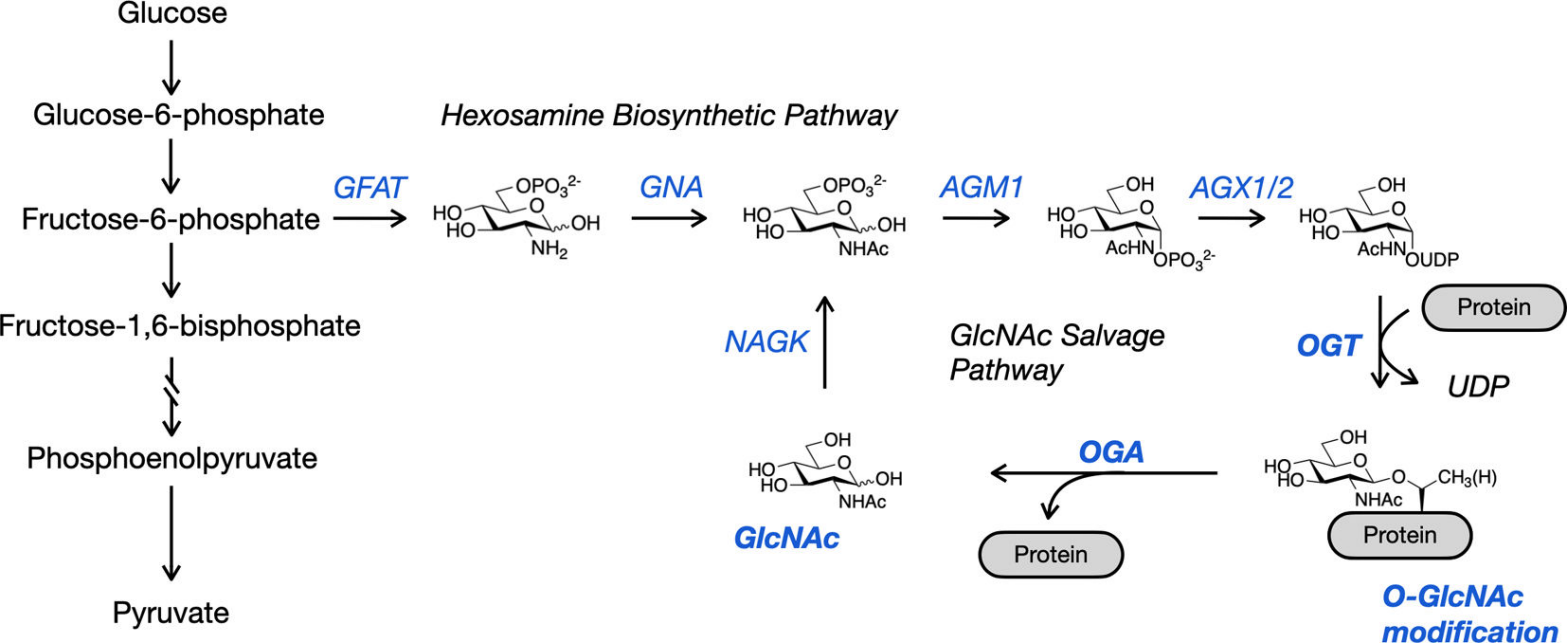
2–3% of glucose entering the cell is diverted from glycolysis into the hexosamine biosynthetic pathway (HBP). The end product of the HBP is UDP-GlcNAc, the high energy donor sugar used by OGT to transfer O-GlcNAc onto protein substrates. When OGA removes O-GlcNAc from proteins, GlcNAc can then by recycled through the GlcNAc salvage pathway which eventually converges with the HBP.

Cells respond to changes in nutrient availability and other environmental signals like growth factors and stress stimuli through metabolic pathways. These pathways are responsible for the production of energy to complete cellular processes and for the synthesis of macromolecules such as lipids, amino acids, carbohydrates and nucleotides. One of these metabolic pathways is the hexosamine biosynthetic pathway (HBP), which utilizes approximately 2–5% of glucose to generate UDP-GlcNAc, the donor sugar for OGT (figure 1) [16]. However, the amount of glucose entering the HBP under stress, disease related conditions, or in different tissues is potentially altered. Importantly, OGT can uniquely respond to changes in UDP-GlcNAc concentrations. OGT has a low apparent *K*_m_ for UDP-GlcNAc, but increasing concentrations of UDP-GlcNAc can change the apparent *K*_m_ for substrates, commonly causing proteins to become better substrates [17]. Thus, an increase of intracellular glucose causes a flux through the HBP, leading to increased UDP-GlcNAc concentrations and more proteins being more extensively O-GlcNAc modified.

Interestingly, the Vocadlo lab shows that inhibition of OGT in mice led to decreased O-GlcNAc levels in various tissues and a corresponding decrease in the satiety-inducing hormone leptin [18]. Similarly, in the hypothalamus, agouti-related protein (AgRP) expressing neurons displayed enriched OGT, and selective knockout of this enzyme resulted in inhibition of neuronal excitability, protecting mice against insulin resistance and diet-induced obesity [19]. These studies highlight the *in vivo* maintenance of metabolic homeostasis and nutrient sensing through modulation of O-GlcNAc levels.

The HBP is an offshoot of glycolysis, where glucose is initially phosphorylated into glucose-6-phosphate and then converted into fructose-6-phosphate. Here, the pathway diverges with fructose-6-phosphate converted into glucosamine-6-phosphate (GlcN-6-P) through the rate limiting enzyme, glutamine fructose-6-phosphate amidotransferase (GFAT). GFAT uses glutamine to catalyse the irreversible addition of the amine to the 2-position of the sugar. Next, glucosamine-6-phosphate N-acetyltransferase (GNA) uses acetyl-CoA to convert GlcN-6-P into GlcNAc-6-phosphate. Then, GlcNAc-6-phosphate is isomerized into GlcNAc-1-phosphate by N-acetylglucosamine mutase (AGM). Finally, UDP-N-acetylglucosamine pyrophosphorylase (AGX1/2) uses UTP and GlcNAc-1-phosphate to generate UDP-GlcNAc.

The rate determining enzyme of the HBP, GFAT, is highly regulated, ensuring that the *de novo* synthesis of UDP-GlcNAc is responsive to other metabolic pathways, nutrient availability and environmental and cellular signals. There are two mammalian isoforms of GFAT, GFAT1 and GFAT2, with close sequence similarity but different tissue expression. GFAT is feedback inhibited by its product glucosamine-6-phosphate, as well as by the HBP end product, UDP-GlcNAc. Regulation of GFAT also occurs at the translational level through mRNA expression levels and at the post-translational level through phosphorylation [20,21]. GFAT is phosphorylated at Ser205 by protein kinase A (PKA), which lowers cellular GFAT activity and abolishes UDP-GlcNAc feedback inhibition [22]. GFAT is also phosphorylated at Ser243 by AMP-activated protein kinase (AMPK), similarly causing a decrease in activity [23]. AMPK activity is regulated by the ratio of ATP:AMP, indicating a direct link between GFAT activity and alterations in cellular energy. This further highlights the complex regulation of the HBP, controlling global O-GlcNAc levels in response to different cellular stimuli.

In addition to the HBP, UDP-GlcNAc can also be generated through a salvage pathway. Glucosamine (GlcN) or N-acetylglucosamine (GlcNAc) can be phosphorylated at the 6-hydroxyl position to generate glucasmine-6-phosphate and N-acetylglucosamine-6-phosphate. These two pathways are interconnected. GlcN or GlcNAc treatment bypasses GFAT to elevate O-GlcNAc levels [24]. In addition, when N-acetylglucosamine kinase (NAGK), the enzyme that phosphorylates GlcNAc, is deleted, de novo HBP synthesis increases [25]. GlcN deprivation also causes a decrease in HBP synthesis and an increase in NAGK dependent salvage [26]. These studies indicate a crosstalk between the HBP and the salvage pathway in responding to nutrient availability.

## 3. O-GlcNAc in diabetes and insulin resistance

Diabetes mellitus is a chronic metabolic disorder characterized by hyperglycaemia, resulting in elevate blood glucose levels. An increase of glucose into the HBP results in higher UDP-GlcNAc concentrations and a corresponding increase in O-GlcNAc modification of proteins. Insulin resistance is a hallmark of type 2 diabetes and is also highly influenced by the flux of the HBP. Thus, the byproduct of the pathway, O-GlcNAc, is heavily implicated in diabetes and insulin resistance. Importantly, several studies have confirmed that O-GlcNAc levels in the cells and tissues of diabetic animals and humans are abnormally increased [27–29].

In 1991, early evidence in primary rat adipocytes showed that elevated UDP-GlcNAc levels were associated with glucose induced insulin resistance [30]. This was further confirmed when transgenic mice overexpressing human OGT in muscle and fat displayed higher O-GlcNAc levels and corresponding insulin resistance and hyperleptinemia [31]. Similarly, knockout of OGA in mice resulted in low circulating glucose, low liver glycogen stores and reduced insulin sensitivity [3]. However, a study by Robinson *et al*. showed that overexpression of OGA or knockdown of OGT did not cause insulin resistance in adipocytes [32]. One explanation for these results is that expression of OGA and OGT are known to be highly dynamic, and manipulation of OGA or OGT levels can cause a reciprocal change in the expression of the other enzyme. Furthermore, inhibition of OGA by PUGNAc showed increased O-GlcNAc levels and impaired insulin signalling in adipocytes and skeletal muscle [33,34]. However, inhibition of OGA with a more selective inhibitor did not replicate the insulin resistance phenotype [35]. This could be due to an off-target effect by the inhibitor PUGNAc, which has been shown to inhibit other hexosaminidases. Despite these conflicting results, the importance of increased O-GlcNAc in diabetes is supported by human genetics, where polymorphisms in the gene encoding OGA results in an increased risk for type II diabetes and a lower age of onset [36]. Ultimately taken together, these data establish a biological role for O-GlcNAc in diabetes and supports further examination into its participation at the mechanistic level.

### 3.1. O-GlcNAc modifies many proteins in insulin signalling pathways

**Figure 2.**
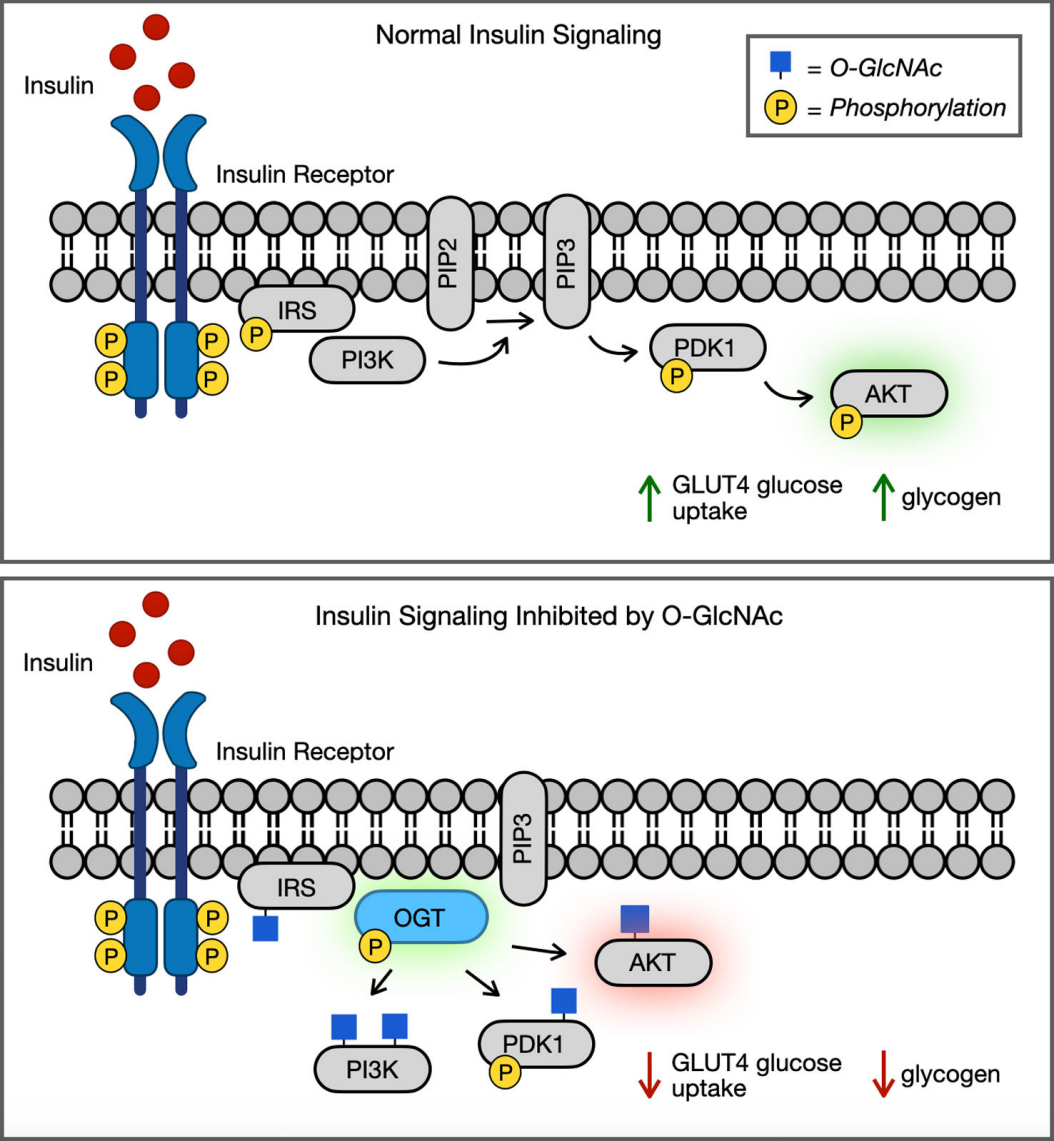
(*a*) Normal insulin signalling and (*b*) insulin signalling inhibited by O-GlcNAc. O-GlcNAc regulates insulin signalling through translocation of OGT to the plasma membrane during prolonged insulin signalling. At the plasma membrane, OGT then modifies multiple proteins including AKT. O-GlcNAcylation of AKT reduces its activity, decreasing the phosphorylation of its substrates and affecting multiple downstream signalling cascades.

Many of the proteins participating in insulin signalling are known to be O-GlcNAc modified, and the crosstalk between O-GlcNAcylation and phosphorylation has been demonstrated to affect several key diabetic signalling pathways. In normal conditions, insulin interacts with its insulin receptor (IR), resulting in autophosphorylation of the IR at multiple tyrosine residues (figure 2) [10,37]. Once activated, the IR recruits and phosphorylates the insulin receptor substrate (IRS1/2). This results in the docking of the p85 subunit of phosphatidyl 3-kinase (PI3K) to the IR/IRS complex. At the plasma membrane, PI3K then catalyses the production of phosphatidylinositol-3,4,5-triphosphate (PIP3) and recruits the PIP3 dependent kinase (PDK1). PDK1 then activates protein kinase B (AKT) through phosphorylation [38]. Active AKT then phosphorylates numerous protein substrates. For example, AKT phosphorylation of Forehead family transcription factors (FOXO) results in decreased transcription of gluconeogenic genes [39]. Also, AKT phosphorylation of Rab-GTPase activating proteins causes insulin responsive glucose transporter GLUT4 to translocate to the plasma membrane. Finally, AKT phosphorylation of glycogen synthase kinase 3β (GSK3β) signals for increased glycogen production.

Upon insulin stimulation, OGT partially translocates to the plasma membrane. There it associates with the IR and undergoes tyrosine phosphorylation, causing an increase in activity and the O-GlcNAcylation of multiple proteins in that signalling pathway [40,41]. O-GlcNAc on AKT reduces phosphorylation at its activation loop on Thr308, the major activating phosphorylation site [29,42]. Phosphorylation of AKT at Ser477 and Thr479 is also activating, and AKT can be further modified by O-GlcNAc at Ser473. Through protein semi-synthesis, AKT phosphorylation at Thr474 and O-GlcNAcylation at Ser473 resulted in a partial increase in AKT activity towards substrates [42]. Interestingly, phosphorylation and O-GlcNAcylation at Ser473 resulted in similar protein substrate phosphorylation.

O-GlcNAc also reduces PDK1 activation, and alters downstream signalling like GLUT4 mediated glucose uptake [38]. In addition, IRS1 is O-GlcNAc modified in a dynamic and time-dependent manner, with peak modification occurring 30 mins after insulin stimulation before rapidly declining [40]. O-GlcNAc of IRS1 results in a decrease of phosphorylation on Tyr608 and reduces the interaction with PI3K and the activation of AKT [41,43,44]. IRS1 O-GlcNAcylation also results in an increase of serine phosphorylation at Ser632 and Ser635, which contributes to the termination of insulin signalling [40]. Furthermore, IRS2, PI3K and PDK1 have also all been shown to be O-GlcNAc modified after prolonged insulin stimulation [43].

### 3.2. O-GlcNAc modification of transcription factors promotes gluconeogenesis

Diabetics suffer from increased gluconeogenesis in the liver, which is considered a major contributor to hyperglycaemia and diabetic organ damage [45]. O-GlcNAc modifies multiple transcription factors that regulate gluconeogenesis. In normal conditions, the forkhead box protein O1, FOXO1, is phosphorylated by AKT, resulting in its nuclear exclusion, reduced transcriptional activity, and targeting it for degradation [46,47]. However, the FOXO1 transcriptional co-activator, PGC-1α, binds to OGT and targets the enzyme to FOXOs, causing increased O-GlcNAcylation. O-GlcNAc modified FOXO1 is localized preferentially in the nucleus and displays increased transcription of gluconeogenic genes [48,49]. Furthermore, host cell factor C1 (HCF-1) recruits OGT to O-GlcNAcylate PGC-1α, which protects it from degradation [27]. Knockdown of OGT and HCF-1 improved glucose homeostasis in diabetic mice [27].

The cAMP response element binding protein (CREB) and its coactivator, CREB regulated transcription coactivator 2 (CRTC2), also induce transcription of gluconeogenic genes. Dentin *et al*. showed that under hyperglycaemia, CRTC2 is O-GlcNAc modified at Ser70 and Ser171, which translocates it to the nucleus where it promotes transcription of gluconeogenic genes. CRTC2 also associates with CREB, which promotes PGC-1α expression and further targets OGT to FOXO1 [50]. OGA overexpression reduces the O-GlcNAcylation of CRTC2 and lowers gluconeogensis in mice [50].

## 4. O-GlcNAc in cancer metabolism

A major hallmark of cancer is the metabolic reprogramming that cells undergo to support rapid cell growth, proliferation and tumourigenesis. The need for increased glucose uptake results in cancer cells undergoing aerobic glycolysis, also known as the Warburg effect, to produce ATP instead of oxidative phosphorylation. While aerobic glycolysis is inefficient, cancer cells undergoing proliferation must consume nutrients to generate metabolites necessary for their rapid growth and invasion. This change in metabolism can influence flux through the HBP and disrupt O-GlcNAcylation. Not surprisingly, numerous studies have highlighted the misregulation of O-GlcNAc in human cancer. For example, many cancer cells and tissues have been observed to display elevated O-GlcNAc levels, specifically in the colon [51,52], breast [53,54], lung [51], prostate [55], pancreas [56,57] and liver [58]. However, the exact mechanisms through which O-GlcNAc contributes to cancer progression is an ongoing point of investigation. Here, several key topics are addressed regarding O-GlcNAc’s contribution to the metabolic reprogramming of cancer cells, the role of O-GlcNAc in modifying oncogenic transcription factors and O-GlcNAc’s regulation of several proteins in the cell cycle.

### 4.1. O-GlcNAc contributes to cancer metabolic reprogramming

Cancer cells change from undergoing oxidative phosphorylation to glycolysis, and this process is driven by many factors including activation of oncogenes, loss of tumour suppressors and tumour cell hypoxia [59,60]. Cancer cells therefore require an increase of glucose uptake in the tumour cell as well as an upregulation of enzymes involved in glycolysis and glucose transport. Interestingly, several glycolytic enzymes are modified by O-GlcNAc to drive this metabolic reprogramming. For example, phosphoglycerate kinase 1 (PGK1), the first ATP-generating enzyme in glycolysis, is O-GlcNAc modified at Thr255 [61]. O-GlcNAc modification activates PDK1 to translocate into the mitochondria where it acts as a kinase to inhibit pyruvate dehydrogenase complex and reduce oxidative phosphorylation [61]. PKG1 O-GlcNAcylation was also observed to be elevated in human colon cancers [61]. Another key glycolytic enzyme modified by O-GlcNAc is an isoform of pyruvate kinase, PKM2 [62]. Cancer cells preferentially express PKM2 and it is a critical regulator of cancer metabolic reprogramming. PKM2 is O-GlcNAc modified at two residues, Thr405 and Thr406, regulating its oligomerization and function and enhancing glycolysis [62,63]. In addition, PKM2 is upregulated by epidermal growth factor (EGF), which plays a crucial role in cell proliferation. EGF stimulates OGT Tyr976 phosphorylation, which promotes the interaction between PKM2 and OGT to up regulate glycolysis [63].

O-GlcNAc also contributes to metabolic reprogramming by increasing the flux through the pentose phosphate pathway. This is advantageous for cancer cells, leading to increased nucleotide production for proliferation and reduced oxidative stress through the generation of NADPH. For example, O-GlcNAc modifies phosphofructokinase 1 (PFK1), a major regulatory enzyme of glycolysis, reducing its activity and promoting flux through the pentose phosphate pathway [64]. PFK1 O-GlcNAcylation was observed in cells exposed to hypoxia and was elevated in several tumour cell lines [64]. Similarly, glucose-6-phosphate dehydrogenase (G6PD), the rate limiting enzyme of the pentose phosphate pathway, is also O-GlcNAc modified in response to hypoxia [65]. O-GlcNAc on G6PD is activating, leading to an increase in glucose flux through the pentose phosphate pathway [65]. Furthermore, G6PD O-GlcNAcylation is elevated in human lung cancers, and blocking O-GlcNAc on G6PD reduces cancer cell proliferation and tumour growth [65].

### 4.2. O-GlcNAc regulates several oncogenic transcription factors

Several studies have highlighted how O-GlcNAc regulation of transcription factors also contributes to the altered metabolic state of cancer cells. Interestingly, O-GlcNAc has been demonstrated to have both a positive and negative effect on the functions of an oncogenic transcription factor. In 1995, the Hart group identified the transcription factor c-myc as being O-GlcNAc modified at Thr58 [66,67]. c-Myc is a transcription factor upregulated in cancer cells and it contributes to the expression of proteins critical to proliferating cells. Specifically, c-Myc induces expression of enzymes involved in glycolysis, cell cycle control, lipid metabolism and glutamine metabolism. c-Myc is phosphorylated by GSK3β at Ser62 before being phosphorylated at Thr58, causing rapid degradation [66,67]. However, O-GlcNAc at Thr58 blocks c-Myc phosphorylation, stabilizing c-Myc against degradation and promoting its target gene expression.

Another transcription factor, HIF-1α, promotes expression of genes contributing to aerobic glycoslysis and is also stabilized by increased O-GlcNAc levels in cancer cells [68]. By contrast, a similar mechanism has been observed in the O-GlcNAc modification of Ser149 of p53, a tumour suppressing protein [40]. p53 phosphorylation at Thr155 leads to its degradation, however O-GlcNAcylation at the nearby Ser149 blocks phosphorylation at Thr155 and stabilizes p53. In this case, O-GlcNAc promotes p53 tumour suppressing activity. Furthermore, loss of p53 leads to activation of the IKK–NF-κB pathway, which plays a role in oncogenesis [69]. However, a component of the IKK complex, IKKβ, is O-GlcNAc modified at Ser733, the same site as an inactivating phosphorylation. O-GlcNAcylation of IKKβ maintains its catalytic activity, allowing transcription factor NF-κB to induce expression of pro-inflammatory tumour development proteins [69].

### 4.3. O-GlcNAc regulates key proteins involved in the cell cycle

To sustain proliferation, cancer cells must bypass cell cycle checkpoints and promote cell cycle progression. Several studies have implicated O-GlcNAc as an important regulator of cell growth and division, and its misregulation has been observed in cancer progression. In mouse embryonic fibroblasts, deletion of OGT was associated with delayed growth, upregulation of the cyclin inhibitor p27 and cell death [2,70]. In addition, decreased UDP-GlcNAc concentrations resulting in decreased O-GlcNAc modifications led to reduced cell division and cell cycle defects [71]. Slawson *et al*. also showed that disruption of O-GlcNAc through pharmacological or genetic manipulation of OGT or OGA resulted in multiple major cell cycle defects and a delay in cell cycle progression [72].

These findings emphasize the global involvement of O-GlcNAc in regulating the cell cycle, and several studies also highlight its misregulation in cancer. In human bladder cancer, reducing hyper O-GlcNAcylation by OGT knockdown decreased the proliferation of bladder cancer cells *in vitro*, as well as tumour growth *in vivo* [73]. They also observed that reducing O-GlcNAc triggered apoptosis, increased autophagy and led to cell cycle arrest [73]. Similarly, RNAi inhibition of OGT in breast cancer cells led to the inhibition of tumour growth both *in vitro* and *in vivo*, decreased cell cycle progression and the upregulation of cell cycle inhibitor p27 (Kip1) [53]. In prostate cancer, reducing O-GlcNAc also led to decreased cancer cell invasion and angiogenesis [55].

Despite its clear participation in the cell cycle, there is still much that is unknown about O-GlcNAc’s specific role. Studies have found some pieces of the puzzle, highlighting several key regulators of the cell cycle that are modified by O-GlcNAc. Specifically, O-GlcNAc modifies cyclin D1, an important regulator of cell cycle entry and G1 phase progression. Cyclin D1 O-GlcNAcylation regulates its half-life and increases stability by modulating its ubiquitination [74,75]. In addition, increased levels of O-GlcNAc modified cyclin D1 resulted in increased proliferation. Another important enzyme of cell cycle progression is Forkhead Box M1 (FoxM1), which upregulates expression of genes involved in G1/S progression. FoxM1 O-GlcNAcylation is also implicated in its stability and reducing O-GlcNAc led to a reduction of FoxM1 levels and increased expression of cell cycle inhibitor p27 (Kip1). Finally, O-GlcNAc also indirectly modulates cyclin-dependent kinase 1 (Cdk1) activity, a regulator of M-phase progression [76]. Cdk1 activity is inhibited by Myelin transcription factor 1 (MYT1) phosphorylation, and it is activated through dephosphorylation by cell division cycle 25C (cdc25) [76]. Hyper O-GlcNAcylation reduces expression of Polo-like kinase 1, an upstream regulator of both MYT1 and cdc25, leading to decreased activity of Cdk1 [76].While still an incomplete picture, these studies highlight the direct and indirect ways hyper O-GlcNAcylation in cancer contributes to the misregulation of the cell cycle, leading to uncontrolled cell proliferation.

## 5. O-GlcNAc in neurodegeneration

Neurodegenerative diseases (NDDs) are chronic and typically late-onset disorders characterized by a loss in motor, sensor and cognitive functions. Here, we will focus on the most common of these NDDs: Alzheimer’s disease (AD) and Parkinson’s disease (PD). Both of these diseases are defined by an early event misfolding of their associated proteins, leading to the formation of amyloid-type aggregates. Specifically, AD is associated with the aggregation of tau and amyloid β peptides, while PD is associated with the aggregation of α-synuclein. While the specific cause and localization of these aggregates varies, the fibrils undergo a progressive prion-like transmission that drives further protein aggregation. The biological consequences of this transmission are complicated and numerous; however, they ultimately result in neuronal death and brain atrophy.

**Figure 3.**
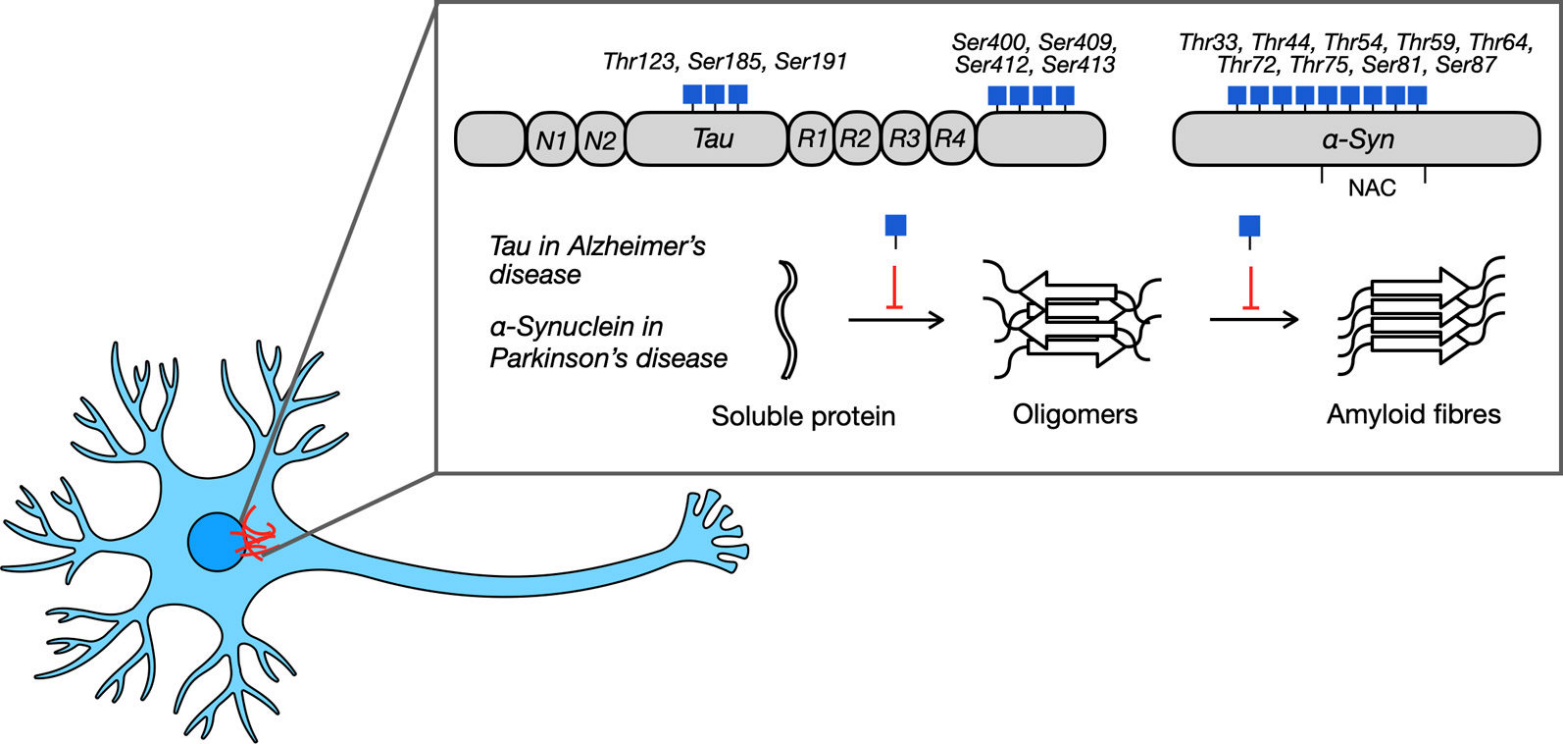
Neurodegenerative diseases like Alzheimer’s disease and Parkinson’s disease are implicated by soluble proteins in neurons, tau and α-Syn, that undergo nucleation to form oligomers. These oligomers then undergo molecular rearrangement and rapid extension to form protein aggregates. O-GlcNAc modifies tau and α-Syn at multiple sites and inhibits the nucleation and extension steps to slow amyloid formation.

Accumulating evidence supports a correlation between neurodegeneration and metabolic impairment. This is not surprising since the brain is the main consumer of glucose, with glucose metabolism driving brain function [77]. For example, insulin dysfunction and severe changes in glucose metabolism was observed in AD and correlated with the severity of cognitive decline [78]. In addition, human corticol neurons were generated from pluripotent stem cells and treated with low glucose media, resulting in decreased O-GlcNAcylation and AD-like changes in corticol neurons [79]. Furthermore, OGT was found to have increased expression and activity in the brain [80]. Neuron specific knockout of OGT results in neuronal defects and progressive neurodegeneration in mice [81,82]. Finally, defects in the chromosome that maps to OGA (10q24.1) is associated with late-onset AD, while defects in the chromosome that maps to OGT (Xq13.1) is associated with X-linked Parkinson’s dystonia [2,83,84]. Taken together, these studies emphasize the link between metabolic processing and O-GlcNAcylation and the onset and development of neurodegeneration. Here, we will review the specific findings investigating O-GlcNAc on the most common NDD proteins: tau in AD and α-synuclein (αSyn) in PD (figure 3).

### 5.1. O-GlcNAc on tau in Alzheimer’s disease

Alzheimer’s disease is the leading cause of dementia and the most common NDD. An early process in AD that contributes to disease progression is the misfolding and aggregation of amyloid beta (Aβ) peptides and Tau. Tau is a highly soluble protein found in the brain, and its primary function is to associate with tubulin to stabilize microtubules and promote tubulin polymerization into microtubules. Tau can oligomerize to form fibrils, which are a major component of neurofibrillary tangles (NFTs), a hallmark of AD. While tau can be phosphorylated, aggregated tau is hyperphosphorylated, and detection of hyperphosphorylated tau is commonly used to detect the presence of NFTs. Tau was also discovered to be O-GlcNAc modified in 1996 by the Hart group in bovine brains at multiple sites [85]. The exact sites of modification were later found to be at Thr123, Ser185, Ser191, Ser400, Ser409, Ser412 and Ser413 [86,87]. Further investigations identified O-GlcNAc at Ser262 in AD brains and confirmed Ser400 in rat brains and JNPL3 AD mouse models [76,88]. Of these sites, Thr123 and Ser400 were identified as the major sites of modification.

Several studies have indicated that O-GlcNAc has a neuroprotective role on tau and can reduce tauopathy. Treatment of PC-12 cells and rat brains with OGA inhibitor thiamet-G led to significantly decreased phosphorylation of tau at pathological sites [89]. When JNPL3 AD mouse models were treated with inhibitor thiamet-G, they also observed reduced tau phosphorylation and a decrease in NFT formation and neuronal death [90]. This was further seen in the rTg4510 mouse, another common AD mouse model, that were treated with thiamet-G and had reduced NFT formation [91]. Importantly, postnatal deletion of OGT in the forebrain neurons of mice resulted in adult mice with progressive neurodegeneration and hyperphosphorylated tau [82]. Studies have also investigated the biochemistry of O-GlcNAc on tau aggregation. A truncated O-GlcNAc modified tau (residues 244–441) containing the amyloid core of tau was coexpressed with OGT, resulting in O-GlcNAc on tau at a mixture of sites and at 50% stoichiometry [92]. This mixture of O-GlcNAc modified tau exhibited slower aggregation than unmodified tau. Mutation of Ser400 to alanine led to a reduction of O-GlcNAc and a loss of the inhibitory effect. Furthermore, the crosstalk between O-GlcNAc and hyperphosphorylation on tau was studied, with O-GlcNAc on Ser400 blocking phosphorylation at Ser396, Ser400 and Ser404 [87]. These results demonstrate that O-GlcNAc can inhibit tau aggregation and prevent tau hyperphosphorylation.

Several companies are investigating pharmacological inhibition of OGA as a therapeutic strategy to treat NDDs, and AD in particular. Merck and Alectos Therapeutics were the first to advance to clinical trials for OGA inhibitor MK-8719, which was well tolerated in single and multiple repeat dosing. Additionally, Asceneuron and Lilly are evaluating ASN90 and LY3372689, two structurally distinct OGA inhibitors. Both are well tolerated and display no serious adverse effects. LY3372689 has been advanced to phase II trials in early symptomatic AD patients. Collectively, these and other OGA inhibitors have been shown to be generally safe in humans, with promising results towards the treatment of NDDs.

### 5.2. O-GlcNAc on α-synuclein in Parkinson’s disease

After AD, Parkinson’s disease (PD) is the second most common form of NDD. An early contributor of PD progression is the aggregation of α-synuclein (α-Syn), a neuronal protein enriched in the brain in presynaptic termini and synaptic vesicles. α-Syn is also the primary component in Lewy bodies, a common pathological marker of PD that induces neuronal toxicity. α-Syn is composed of 140 amino acids, with residues 61–95 named the non-amyloid component when it was first identified as a peptide in AD aggregates. This region of α-Syn was later confirmed to be essential for driving its fibril aggregation. Furthermore, α-Syn fibrils have been implicated in multiple other NDDs, termed synucleinopathies, including PD, multiple systems atrophy (MSA), and dementia with Lewy bodies. Similar to tau in AD, multiple studies have indicated that O-GlcNAc on α-Syn plays a neuroprotective role by attenuating its pathological aggregation. α-Syn has been identified in mouse and human brain tissues to be site specifically modified by O-GlcNAc at nine different sites [1,76,84,93–95]. Interestingly, five of these modifications are located within the non-amyloid component of α-Syn, the core region responsible for driving amyloid aggregation. This further implicates O-GlcNAc’s potential in modulating the aggregation of α-Syn monomers.

Knockdown of OGA by RNAi or through thiamet-G inhibition in both neuroblastoma cells and primary neurons resulted in decreased uptake of α-Syn preformed fibrils [96]. In mice, thiamet-G was delivered with an adeno-associated virus driving overexpression of A53T α-Syn, a mutant prone to aggregation [97]. This resulted in decreased pathology and neurodegeneration. Furthermore, OGA inhibition with ASN90 in transgenic mice overexpressing human α-Syn led to increased O-GlcNAcylation, reduced astrogliosis and slowed the progression of motor impairment [98]. There studies support further investigation into the use of OGA inhibitors for the treatment of synucleinopathies.

The site-specific effects of O-GlcNAc on α-Syn have been interrogated in several important studies by our group. O-GlcNAc on Thr72 was shown to inhibit amyloid formation by slowing the nucleation step of aggregation, however it did not inhibit further extension [99]. In a follow up study, O-GlcNAc on Ser87 was similarly found to effect α-Syn nucleation [100], although interestingly the modification on Ser87 and Thr72 had site specific differences on the extent of inhibition, with Thr72 exhibiting slower kinetics. These two sites of modification were also shown to inhibit proteolytic cleavage of α-Syn by calpain, preventing the truncation fragments from aggregating [101]. O-GlcNAc on Thr72, Thr75, Thr81 and Ser87 were also characterized and found to similarly reduce amyloid aggregation, with O-GlcNAc on Thr72 and Thr81 displaying the highest inhibitory effect [102]. Notably, it was observed that modification of Thr72 and Ser87 caused the formation of a different structure compared with unmodified α-Syn. This result was further explored when the α-Syn fibrils of O-GlcNAc modified Ser87 were shown to seed aggregation of unmodified α-Syn *in vitro,* however when added to neurons or mouse brains the fibrils lowered seeding and pathogenicity [103]. Collectively, these studies support a direct role of O-GlcNAc in PD by emphasizing the site-specific effects of the modification in slowing α-Syn aggregation and promoting fibril structures that are less pathogenetic.

## 6. Conclusion

Cellar metabolism and the modification O-GlcNAc are intimately linked. Through the HBP and the synthesis of UDP-GlcNAc, cells can respond to changes in the environment to control various downstream cellular processes. Perturbed O-GlcNAcylation is a key mechanism in human disease, contributing to disruptions in protein activity and signalling cascades. With diseases like diabetes, cancer and neurodegeneration increasingly becoming major health risks, understanding their aberrant O-GlcNAcylation and the mechanisms that contribute to disease progression will provide better opportunities for treatment. Indeed, OGT and OGA have already been attractive candidates for targeting pharmacologically. However, future work should further investigate the cellular effects of extended increased or decreased O-GlcNAc levels to better understand the consequences of therapeutically inhibiting OGT or OGA. There are also many O-GlcNAc modified protein substrates and signalling pathways that have yet to be discovered and characterized. Overall, these studies provide us with an important foundation in understanding the role of O-GlcNAc in cellular metabolism and disease progression.

## Data Availability

This article has no additional data.

## References

[1] Wang S *et al*. 2012 Extensive crosstalk between O-GlcNAcylation and phosphorylation regulates Akt signaling. PLoS One **7**, e37427. (10.1371/journal.pone.0037427) 22629392 PMC3358304

[2] Shafi R, Iyer SP, Ellies LG, O’Donnell N, Marek KW, Chui D, Hart GW, Marth JD. 2000 The O-GlcNAc transferase gene resides on the X chromosome and is essential for embryonic stem cell viability and mouse ontogeny. Proc. Natl Acad. Sci. USA **97**, 5735–5739. (10.1073/pnas.100471497) 10801981 PMC18502

[3] Keembiyehetty C, Love DC, Harwood KR, Gavrilova O, Comly ME, Hanover JA. 2015 Conditional knock-out reveals a requirement for O-linked N-Acetylglucosaminase (O-GlcNAcase) in metabolic homeostasis. J. Biol. Chem. **290**, 7097–7113. (10.1074/jbc.M114.617779) 25596529 PMC4358131

[4] Ma Z, Vosseller K. 2013 O-GlcNAc in cancer biology. Amino Acids **45**, 719–733. (10.1007/s00726-013-1543-8) 23836420

[5] Hanover JA, Chen W, Bond MR. 2018 O-GlcNAc in cancer: an oncometabolism-fueled vicious cycle. J. Bioenerg. Biomembr. **50**, 155–173. (10.1007/s10863-018-9751-2) 29594839

[6] Hardivillé S, Hart GW. 2014 Nutrient regulation of signaling, transcription, and cell physiology by O-GlcNAcylation. Cell Metab. **20**, 208–213. (10.1016/j.cmet.2014.07.014) 25100062 PMC4159757

[7] Hart GW. 2019 Nutrient regulation of signaling and transcription. J. Biol. Chem. **294**, 2211–2231. (10.1074/jbc.AW119.003226) 30626734 PMC6378989

[8] Liu F, Shi J, Tanimukai H, Gu J, Gu J, Grundke-Iqbal I, Iqbal K, Gong CX. 2009 Reduced O-GlcNAcylation links lower brain glucose metabolism and tau pathology in Alzheimer’s disease. Brain **132**, 1820–1832. (10.1093/brain/awp099) 19451179 PMC2702834

[9] Pinho TS, Correia SC, Perry G, Ambrósio AF, Moreira PI. 2019 Diminished O-GlcNAcylation in Alzheimer’s disease is strongly correlated with mitochondrial anomalies. Biochim. Biophys. Acta. Mol. Basis Dis. **1865**, 2048–2059. (10.1016/j.bbadis.2018.10.037) 30412792

[10] Slawson C, Copeland RJ, Hart GW. 2010 O-GlcNAc signaling: a metabolic link between diabetes and cancer? Trends Biochem. Sci. **35**, 547–555. (10.1016/j.tibs.2010.04.005) 20466550 PMC2949529

[11] Ruan HB, Singh JP, Li MD, Wu J, Yang X. 2013 Cracking the O-GlcNAc code in metabolism. Trends Endocrinol. Metab. **24**, 301–309. (10.1016/j.tem.2013.02.002) 23647930 PMC3783028

[12] Bond MR, Hanover JA. 2013 O-GlcNAc cycling: a link between metabolism and chronic disease. Annu. Rev. Nutr. **33**, 205–229. (10.1146/annurev-nutr-071812-161240) 23642195 PMC10483992

[13] Ma J, Hou C, Wu C. 2022 Demystifying the O-GlcNAc Code: a systems view. Chem. Rev. **122**, 15822–15864. (10.1021/ACS.CHEMREV.1C01006/SUPPL_FILE/CR1C01006_SI_002.XLSX) 35302357

[14] Fehl C, Hanover JA. 2022 Tools, tactics and objectives to interrogate cellular roles of O-GlcNAc in disease. Nat. Chem. Biol. **18**, 8–17. (10.1038/s41589-021-00903-6) 34934185 PMC8712397

[15] Pratt MR, Vocadlo DJ. 2023 Understanding and exploiting the roles of O-GlcNAc in neurodegenerative diseases. J. Biol. Chem. **299**, 105411. (10.1016/j.jbc.2023.105411) 37918804 PMC10687168

[16] Marshall S, Bacote V, Traxinger RR. 1991 Discovery of a metabolic pathway mediating glucose-induced desensitization of the glucose transport system. Role of hexosamine biosynthesis in the induction of insulin resistance. J. Biol. Chem. **266**, 4706–4712. (10.1016/S0021-9258(19)67706-9) 2002019

[17] Haltiwanger RS, Blomberg MA, Hart GW. 1992 Glycosylation of nuclear and cytoplasmic proteins. Purification and characterization of a uridine diphospho-N-acetylglucosamine:polypeptide beta-N-acetylglucosaminyltransferase. J. Biol. Chem. **267**, 9005–9013. (10.1016/S0021-9258(19)50380-5) 1533623

[18] Liu TW, Zandberg WF, Gloster TM, Deng L, Murray KD, Shan X, Vocadlo DJ. 2018 Metabolic inhibitors of O-GlcNAc transferase that act in vivo implicate decreased O-GlcNAc levels in leptin-mediated nutrient sensing. Angew. Chem. Int. Ed. **57**, 7644–7648. (10.1002/anie.201803254) PMC6055616 29756380

[19] Ruan HB *et al*. 2014 O-GlcNAc transferase enables AgRP neurons to suppress browning of white fat. Cell **159**, 306–317. (10.1016/j.cell.2014.09.010) 25303527 PMC4509746

[20] Yki-Järvinen H, Daniels MC, Virkamäki A, Mäkimattila S, DeFronzo RA, McClain D. 1996 Increased glutamine:fructose-6-phosphate amidotransferase activity in skeletal muscle of patients with NIDDM. Diabetes **45**, 302–307. (10.2337/diab.45.3.302) 8593934

[21] Srinivasan V, Sandhya N, Sampathkumar R, Farooq S, Mohan V, Balasubramanyam M. 2007 Glutamine fructose-6-phosphate amidotransferase (GFAT) gene expression and activity in patients with type 2 diabetes: inter-relationships with hyperglycaemia and oxidative stress. Clin. Biochem. **40**, 952–957. (10.1016/j.clinbiochem.2007.05.002) 17574229

[22] Ruegenberg S, Mayr FAMC, Atanassov I, Baumann U, Denzel MS. 2021 Protein kinase A controls the hexosamine pathway by tuning the feedback inhibition of GFAT-1. Nat. Commun. **12**, 2176. (10.1038/s41467-021-22320-y) 33846315 PMC8041777

[23] Eguchi S, Oshiro N, Miyamoto T, Yoshino KI, Okamoto S, Ono T, Kikkawa U, Yonezawa K. 2009 AMP-activated protein kinase phosphorylates glutamine: fructose-6-phosphate amidotransferase 1 at Ser243 to modulate its enzymatic activity. Gene. Cell. **14**, 179–189. (10.1111/j.1365-2443.2008.01260.x) 19170765

[24] Hresko RC, Heimberg H, Chi MM, Mueckler M. 1998 Glucosamine-induced insulin resistance in 3T3-L1 adipocytes is caused by depletion of intracellular ATP. J. Biol. Chem. **273**, 20658–20668. (10.1074/jbc.273.32.20658) 9685425

[25] Campbell S *et al*. 2021 Glutamine deprivation triggers NAGK-dependent hexosamine salvage. eLife **10**, e62644. (10.7554/eLife.62644) 34844667 PMC8631944

[26] Kim PK *et al*. 2021 Hyaluronic acid fuels pancreatic cancer cell growth. eLife **10**, e62645. (10.7554/eLife.62645) 34951587 PMC8730721

[27] Ruan HB *et al*. 2012 O-GlcNAc transferase/host cell factor C1 complex regulates gluconeogenesis by modulating PGC-1α stability. Cell Metab. **16**, 226–237. (10.1016/j.cmet.2012.07.006) 22883232 PMC3480732

[28] Fricovsky ES *et al*. 2012 Excess protein O-GlcNAcylation and the progression of diabetic cardiomyopathy. Am. J. Physiol. Regul. Integr. Comp. Physiol. **303**, R689–99. (10.1152/ajpregu.00548.2011) 22874425 PMC3469670

[29] Walgren JLE, Vincent TS, Schey KL, Buse MG. 2003 High glucose and insulin promote O-GlcNAc modification of proteins, including α-tubulin. Am. J. Physiol. Endocrinol. Metab. **284**, 424–434. (10.1152/AJPENDO.00382.2002/ASSET/IMAGES/LARGE/H10231166007.JPEG) 12397027

[30] Traxinger RR, Marshall S. 1991 Coordinated regulation of glutamine:fructose-6-phosphate amidotransferase activity by insulin, glucose, and glutamine: role of hexosamine biosynthesis in enzyme regulation. J. Biol. Chem. **266**, 10148–10154. (10.1016/S0021-9258(18)99202-1) 2037571

[31] McClain DA, Lubas WA, Cooksey RC, Hazel M, Parker GJ, Love DC, Hanover JA. 2002 Altered glycan-dependent signaling induces insulin resistance and hyperleptinemia. Proc. Natl Acad. Sci. USA **99**, 10695–10699. (10.1073/PNAS.152346899/ASSET/0D72B65A-239A-406E-9FDC-064CF55A165D/ASSETS/GRAPHIC/PQ1523468004.JPEG) 12136128 PMC125016

[32] Robinson KA, Ball LE, Buse MG. 2007 Reduction of O-GlcNAc protein modification does not prevent insulin resistance in 3T3-L1 adipocytes. Am. J. Physiol. Endocrinol. Metab. **292**, E884–90. (10.1152/ajpendo.00569.2006) 17122093 PMC2366901

[33] Arias EB, Kim J, Cartee GD. 2004 Prolonged incubation in PUGNAc results in increased protein O-Linked glycosylation and insulin resistance in rat skeletal muscle. Diabetes **53**, 921–930. (10.2337/diabetes.53.4.921) 15047606

[34] Vosseller K, Wells L, Lane MD, Hart GW. 2002 Elevated nucleocytoplasmic glycosylation by O-GlcNAc results in insulin resistance associated with defects in Akt activation in 3T3-L1 adipocytes. Proc. Natl Acad. Sci. USA **99**, 5313–5318. (10.1073/pnas.072072399) 11959983 PMC122766

[35] Macauley MS, Bubb AK, Martinez-Fleites C, Davies GJ, Vocadlo DJ. 2008 Elevation of global O-GlcNAc levels in 3T3-L1 adipocytes by selective inhibition of O-GlcNAcase does not induce insulin resistance. J. Biol. Chem. **283**, 34687–34695. (10.1074/jbc.M804525200) 18842583 PMC3259902

[36] Lehman DM *et al*. 2005 A single nucleotide polymorphism in MGEA5 encoding O-GlcNAc-selective N-acetyl-beta-D glucosaminidase is associated with type 2 diabetes in Mexican Americans. Diabetes **54**, 1214–1221. (10.2337/diabetes.54.4.1214) 15793264

[37] Zachara N, Akimoto Y, Hart GW *et al*. 2017 The O-glcnac modification. In Essentials of glycobiology (ed. A Varki), 3rd edn. Woodbury, NY: Cold Spring Harbor Laboratory Press.

[38] Sarbassov DD, Guertin DA, Ali SM, Sabatini DM. 2005 Phosphorylation and regulation of Akt/PKB by the rictor-mTOR complex. Science **307**, 1098–1101. (10.1126/science.1106148) 15718470

[39] Lin Y, Sun Z. 2010 Current views on type 2 diabetes. J. Endocrinol. **204**, 1–11. (10.1677/JOE-09-0260) 19770178 PMC2814170

[40] Yang X *et al*. 2008 Phosphoinositide signalling links O-GlcNAc transferase to insulin resistance. Nature **451**, 964–969. (10.1038/nature06668) 18288188

[41] Whelan SA, Dias WB, Thiruneelakantapillai L, Lane MD, Hart GW. 2010 Regulation of insulin receptor substrate 1 (IRS-1)/AKT kinase-mediated insulin signaling by O-Linked beta-N-acetylglucosamine in 3T3-L1 adipocytes. J. Biol. Chem. **285**, 5204–5211. (10.1074/jbc.M109.077818) 20018868 PMC2820748

[42] Salguero AL *et al*. 2022 Multifaceted regulation of Akt by diverse C-terminal post-translational modifications. ACS Chem. Biol. **17**, 68–76. (10.1021/ACSCHEMBIO.1C00632/ASSET/IMAGES/LARGE/CB1C00632_0006.JPEG) 34941261 PMC8864695

[43] Whelan SA, Lane MD, Hart GW. 2008 Regulation of the O-linked beta-N-acetylglucosamine transferase by insulin signaling. J. Biol. Chem. **283**, 21411–21417. (10.1074/jbc.M800677200) 18519567 PMC2490780

[44] Klein AL, Berkaw MN, Buse MG, Ball LE. 2009 O-linked N-acetylglucosamine modification of insulin receptor substrate-1 occurs in close proximity to multiple SH2 domain binding motifs. Mol. Cell Proteomics **8**, 2733–2745. (10.1074/mcp.M900207-MCP200) 19671924 PMC2816021

[45] Hatting M, Tavares CDJ, Sharabi K, Rines AK, Puigserver P. 2018 Insulin regulation of gluconeogenesis. Ann. N. Y. Acad. Sci. **1411**, 21–35. (10.1111/nyas.13435) 28868790 PMC5927596

[46] Biggs WH, Meisenhelder J, Hunter T, Cavenee WK, Arden KC. 1999 Protein kinase B/Akt-mediated phosphorylation promotes nuclear exclusion of the winged helix transcription factor FKHR1. Proc. Natl Acad. Sci. USA **96**, 7421–7426. (10.1073/pnas.96.13.7421) 10377430 PMC22101

[47] Matsuzaki H, Daitoku H, Hatta M, Tanaka K, Fukamizu A. 2003 Insulin-induced phosphorylation of FKHR (Foxo1) targets to proteasomal degradation. Proc. Natl Acad. Sci. USA **100**, 11285–11290. (10.1073/pnas.1934283100) 13679577 PMC208749

[48] Housley MP, Rodgers JT, Udeshi ND, Kelly TJ, Shabanowitz J, Hunt DF, Puigserver P, Hart GW. 2008 O-GlcNAc regulates FoxO activation in response to glucose. J. Biol. Chem. **283**, 16283–16292. (10.1074/jbc.M802240200) 18420577 PMC2423255

[49] Housley MP, Udeshi ND, Rodgers JT, Shabanowitz J, Puigserver P, Hunt DF, Hart GW. 2009 A PGC-1alpha-O-GlcNAc transferase complex regulates FoxO transcription factor activity in response to glucose. J. Biol. Chem. **284**, 5148–5157. (10.1074/jbc.M808890200) 19103600 PMC2643526

[50] Dentin R, Hedrick S, Xie J, Yates J, Montminy M. 2008 Hepatic glucose sensing via the CREB coactivator CRTC2. Science **391**, 1402–1405. (10.1126/SCIENCE.1151363/SUPPL_FILE/DENTIN_SOM.PDF) 18323454

[51] Mi W, Gu Y, Han C, Liu H, Fan Q, Zhang X, Cong Q, Yu W. 2011 O-GlcNAcylation is a novel regulator of lung and colon cancer malignancy. Biochim. Biophys. Acta **1812**, 514–519. (10.1016/j.bbadis.2011.01.009) 21255644

[52] Yehezkel G, Cohen L, Kliger A, Manor E, Khalaila I. 2012 O-linked β-N-acetylglucosaminylation (O-GlcNAcylation) in primary and metastatic colorectal cancer clones and effect of N-acetyl-β-D-glucosaminidase silencing on cell phenotype and transcriptome. J. Biol. Chem. **287**, 28755–28769. (10.1074/jbc.M112.345546) 22730328 PMC3436545

[53] Caldwell SA, Jackson SR, Shahriari KS, Lynch TP, Sethi G, Walker S, Vosseller K, Reginato MJ. 2010 Nutrient sensor O-GlcNAc transferase regulates breast cancer tumorigenesis through targeting of the oncogenic transcription factor FoxM1. Oncogene **29**, 2831–2842. (10.1038/onc.2010.41) 20190804

[54] Gu Y *et al*. 2010 GlcNAcylation plays an essential role in breast cancer metastasis. Cancer Res. **70**, 6344–6351. (10.1158/0008-5472.CAN-09-1887) 20610629

[55] Lynch TP, Ferrer CM, Jackson SRE, Shahriari KS, Vosseller K, Reginato MJ. 2012 Critical role of O-Linked β-N-acetylglucosamine transferase in prostate cancer invasion, angiogenesis, and metastasis. J. Biol. Chem. **287**, 11070–11081. (10.1074/jbc.M111.302547) 22275356 PMC3322861

[56] Qian K *et al*. 2018 Transcriptional regulation of O-GlcNAc homeostasis is disrupted in pancreatic cancer. J. Biol. Chem. **293**, 13989–14000. (10.1074/JBC.RA118.004709/ATTACHMENT/BFD75DE2-5135-460B-9F29-269E6C75281A/MMC1.PDF) 30037904 PMC6130940

[57] Zhu Q *et al*. 2022 O-GlcNAcylation promotes pancreatic tumor growth by regulating malate dehydrogenase 1. Nat. Chem. Biol. **18**, 1087–1095. (10.1038/s41589-022-01085-5) 35879546

[58] Zhu Q, Zhou L, Yang Z, Lai M, Xie H, Wu L, Xing C, Zhang F, Zheng S. 2012 O-GlcNAcylation plays a role in tumor recurrence of hepatocellular carcinoma following liver transplantation. Med. Oncol. **29**, 985–993. (10.1007/s12032-011-9912-1) 21461968

[59] Zheng J. 2012 Energy metabolism of cancer: Glycolysis versus oxidative phosphorylation (Review). Oncol. Lett. **4**, 1151–1157. (10.3892/ol.2012.928) 23226794 PMC3506713

[60] Ma Z, Vosseller K. 2014 Cancer metabolism and elevated O-GlcNAc in oncogenic signaling. J. Biol. Chem. **289**, 34457–34465. (10.1074/jbc.R114.577718) 25336642 PMC4263853

[61] Nie H, Ju H, Fan J, Shi X, Cheng Y, Cang X, Zheng Z, Duan X, Yi W. 2020 O-GlcNAcylation of PGK1 coordinates glycolysis and TCA cycle to promote tumor growth. Nat. Commun. **11**, 1–14. (10.1038/s41467-019-13601-8) 31911580 PMC6946671

[62] Wang Y *et al*. 2017 O-GlcNAcylation destabilizes the active tetrameric PKM2 to promote the Warburg effect. Proc. Natl Acad. Sci. USA **114**, 13732–13737. (10.1073/PNAS.1704145115/SUPPL_FILE/PNAS.1704145115.SM01.MOV) 29229835 PMC5748163

[63] Wang Y *et al*. 2022 EGF promotes PKM2 O-GlcNAcylation by stimulating O-GlcNAc transferase phosphorylation at Y976 and their subsequent association. J. Biol. Chem. **298**, 102340. (10.1016/j.jbc.2022.102340) 35931120 PMC9436816

[64] Yi W, Clark PM, Mason DE, Keenan MC, Hill C, Goddard WA, Peters EC, Driggers EM, Hsieh-Wilson LC. 2012 Phosphofructokinase 1 glycosylation regulates cell growth and metabolism. Science **337**, 975–980. (10.1126/SCIENCE.1222278/SUPPL_FILE/YI.W.SM.PDF) 22923583 PMC3534962

[65] Rao X *et al*. 2015 O-GlcNAcylation of G6PD promotes the pentose phosphate pathway and tumor growth. Nat. Commun. **6**, 8468. (10.1038/ncomms9468) 26399441 PMC4598839

[66] Chou TY, Harts GW, Lane MD. 1995 Glycosylation of the c-Myc transactivation domain (O-linked N-acetylglucosamine/nuclear glycosylation/protein-protein interaction/protooncogene). Biochemistry **92**, 4417–4421. (10.1073/pnas.92.10.4417) PMC41955 7753821

[67] Chou TY, Hart GW, Dang CV. 1995 c-Myc is glycosylated at threonine 58, a known phosphorylation site and a mutational hot spot in lymphomas. J. Biol. Chem. **270**, 18961–18965. (10.1074/jbc.270.32.18961) 7642555

[68] Ferrer CM, Lynch TP, Sodi VL, Falcone JN, Schwab LP, Peacock DL, Vocadlo DJ, Seagroves TN, Reginato MJ. 2014 O-GlcNAcylation regulates cancer metabolism and survival stress signaling via regulation of the HIF-1 pathway. Mol. Cell **54**, 820–831. (10.1016/j.molcel.2014.04.026) 24857547 PMC4104413

[69] Kawauchi K, Araki K, Tobiume K, Tanaka N. 2009 Loss of p53 enhances catalytic activity of IKKβ through O-linked β-N-acetyl glucosamine modification. Proc. Natl Acad. Sci. USA **106**, 3431–3436. (10.1073/PNAS.0813210106/SUPPL_FILE/0813210106SI.PDF) 19202066 PMC2651314

[70] O’Donnell N, Zachara NE, Hart GW, Marth JD. 2004 Ogt-dependent X-chromosome-linked protein glycosylation is a requisite modification in somatic cell function and embryo viability. Mol. Cell. Biol. **24**, 1680–1690. (10.1128/MCB.24.4.1680-1690.2004) 14749383 PMC344186

[71] Boehmelt G *et al*. 2000 Decreased UDP-GlcNAc levels abrogate proliferation control in EMeg32-deficient cells. EMBO J. **19**, 5092–5104. (10.1093/emboj/19.19.5092) 11013212 PMC302091

[72] Slawson C, Zachara NE, Vosseller K, Cheung WD, Lane MD, Hart GW. 2005 Perturbations in O-linked beta-N-acetylglucosamine protein modification cause severe defects in mitotic progression and cytokinesis. J. Biol. Chem. **280**, 32944–32956. (10.1074/jbc.M503396200) 16027160

[73] Wang L *et al*. 2018 Suppressed OGT expression inhibits cell proliferation while inducing cell apoptosis in bladder cancer. BMC Cancer **18**, 1–12. (10.1186/S12885-018-5033-Y/FIGURES/6) 30453909 PMC6245611

[74] Masclef L, Dehennaut V, Mortuaire M, Schulz C, Leturcq M, Lefebvre T, Vercoutter-Edouart AS. 2019 Cyclin D1 stability is partly controlled by O-GlcNAcylation. Front. Endocrinol. (Lausanne) **10**, 106. (10.3389/fendo.2019.00106) 30853938 PMC6395391

[75] Olivier-Van Stichelen S, Drougat L, Dehennaut V, El Yazidi-Belkoura I, Guinez C, Mir AM, Michalski JC, Vercoutter-Edouart AS, Lefebvre T. 2012 Serum-stimulated cell cycle entry promotes ncOGT synthesis required for cyclin D expression. Oncogenesis **1**, e36. (10.1038/oncsis.2012.36) 23552487 PMC3545199

[76] Wang Z, Udeshi ND, O’Malley M, Shabanowitz J, Hunt DF, Hart GW. 2010 Enrichment and site mapping of O-linked N-acetylglucosamine by a combination of chemical/enzymatic tagging, photochemical cleavage, and electron transfer dissociation mass spectrometry. Mol. Cell Proteomics **9**, 153–160. (10.1074/mcp.M900268-MCP200) 19692427 PMC2808261

[77] Mergenthaler P, Lindauer U, Dienel GA, Meisel A. 2013 Sugar for the brain: the role of glucose in physiological and pathological brain function. Trends Neurosci. **36**, 587–597. (10.1016/j.tins.2013.07.001) 23968694 PMC3900881

[78] Heiss WD, Szelies B, Kessler J, Herholz K. 1991 Abnormalities of energy metabolism in Alzheimer’s disease studied with PET. Ann. N. Y. Acad. Sci. **640**, 65–71. (10.1111/j.1749-6632.1991.tb00192.x) 1776760

[79] Huang CW, Rust NC, Wu HF, Yin A, Zeltner N, Yin H, Hart GW. 2023 Low glucose induced Alzheimer’s disease‐like biochemical changes in human induced pluripotent stem cell‐derived neurons is due to dysregulated O‐GlcNAcylation. Alzheimers. Dement. **19**, 4872–4885. (10.1002/alz.13058) 37037474 PMC10562522

[80] Akimoto Y, Comer FI, Cole RN, Kudo A, Kawakami H, Hirano H, Hart GW. 2003 Localization of the O-GlcNAc transferase and O-GlcNAc-modified proteins in rat cerebellar cortex. Brain Res. **966**, 194–205. (10.1016/s0006-8993(02)04158-6) 12618343

[81] Su C, Schwarz TL. 2017 O-GlcNAc transferase is essential for sensory neuron survival and maintenance. J. Neurosci. **37**, 2125–2136. (10.1523/JNEUROSCI.3384-16.2017) 28115479 PMC5338757

[82] Wang AC, Jensen EH, Rexach JE, Vinters HV, Hsieh-Wilson LC. 2016 Loss of O-GlcNAc glycosylation in forebrain excitatory neurons induces neurodegeneration. Proc. Natl Acad. Sci. USA **113**, 15120–15125. (10.1073/pnas.1606899113) 27956640 PMC5206508

[83] Deng Y, Li B, Liu Y, Iqbal K, Grundke-Iqbal I, Gong CX. 2009 Dysregulation of insulin signaling, glucose transporters, O-GlcNAcylation, and phosphorylation of tau and neurofilaments in the brain: implication for Alzheimer’s disease. Am. J. Pathol. **175**, 2089–2098. (10.2353/ajpath.2009.090157) 19815707 PMC2774072

[84] Willems AP *et al*. 2017 Mutations in N-acetylglucosamine (O-GlcNAc) transferase in patients with X-linked intellectual disability. J. Biol. Chem. **292**, 12621–12631. (10.1074/jbc.M117.790097) 28584052 PMC5535036

[85] Shane Arnold C, W Johnson GV, Cole RN, L-Y Dong D, Lee M, Hart GW. 1996 The microtubule-associated protein tau is extensively modified with O-linked N-acetylglucosamine. J. Biol. Chem. **271**, 28741–28744. (10.1074/jbc.271.46.28741) 8910513

[86] Yuzwa SA, Yadav AK, Skorobogatko Y, Clark T, Vosseller K, Vocadlo DJ. 2011 Mapping O-GlcNAc modification sites on tau and generation of a site-specific O-GlcNAc tau antibody. Amino Acids **40**, 857–868. (10.1007/s00726-010-0705-1) 20706749

[87] Bourré G, Cantrelle FX, Kamah A, Chambraud B, Landrieu I, Smet-Nocca C. 2018 Direct crosstalk between O-GlcNAcylation and phosphorylation of tau protein investigated by NMR spectroscopy. Front. Endocrinol. (Lausanne) **9**, 595. (10.3389/fendo.2018.00595) 30386294 PMC6198643

[88] Morris M, Knudsen GM, Maeda S, Trinidad JC, Ioanoviciu A, Burlingame AL, Mucke L. 2015 Tau post-translational modifications in wild-type and human amyloid precursor protein transgenic mice. Nat. Neurosci. **18**, 1183–1189. (10.1038/nn.4067) 26192747 PMC8049446

[89] Yuzwa SA *et al*. 2008 A potent mechanism-inspired O-GlcNAcase inhibitor that blocks phosphorylation of tau in vivo. Nat. Chem. Biol. **4**, 483–490. (10.1038/nchembio.96) 18587388

[90] Lewis J *et al*. 2000 Neurofibrillary tangles, amyotrophy and progressive motor disturbance in mice expressing mutant (P301L) tau protein. Nat. Genet. **25**, 402–405. (10.1038/78078) 10932182

[91] Graham DL, Gray AJ, Joyce JA, Yu D, O’Moore J, Carlson GA, Shearman MS, Dellovade TL, Hering H. 2014 Increased O-GlcNAcylation reduces pathological tau without affecting its normal phosphorylation in a mouse model of tauopathy. Neuropharmacology **79**, 307–313. (10.1016/j.neuropharm.2013.11.025) 24326295

[92] Yuzwa SA, Shan X, Macauley MS, Clark T, Skorobogatko Y, Vosseller K, Vocadlo DJ. 2012 Increasing O-GlcNAc slows neurodegeneration and stabilizes tau against aggregation. Nat. Chem. Biol. **8**, 393–399. (10.1038/nchembio.797) 22366723

[93] Wang Z, Park K, Comer F, Hsieh-Wilson LC, Saudek CD, Hart GW. 2009 Site-specific GlcNAcylation of human erythrocyte proteins: potential biomarker(s) for diabetes. Diabetes **58**, 309–317. (10.2337/db08-0994) 18984734 PMC2628603

[94] Alfaro JF *et al*. 2012 Tandem mass spectrometry identifies many mouse brain O-GlcNAcylated proteins including EGF domain-specific O-GlcNAc transferase targets. Proc. Natl Acad. Sci. USA **109**, 7280–7285. (10.1073/pnas.1200425109) 22517741 PMC3358849

[95] Huynh VN *et al*. 2021 Defining the dynamic regulation of O-GlcNAc proteome in the mouse cortex---the O-GlcNAcylation of synaptic and trafficking proteins related to neurodegenerative diseases. Front. Aging **2**. (10.3389/fragi.2021.757801) PMC9261315 35822049

[96] Tavassoly O, Yue J, Vocadlo DJ. 2021 Pharmacological inhibition and knockdown of O‐GlcNAcase reduces cellular internalization of α‐synuclein preformed fibrils. FEBS J. **288**, 452–470. (10.1111/febs.15349) 32365408

[97] Lee BE *et al*. 2020 O- GlcNAcylation regulates dopamine neuron function, survival and degeneration in Parkinson disease . Brain **143**, 3699–3716. (10.1093/brain/awaa320) 33300544 PMC7805798

[98] Permanne B, Sand A, Ousson S, Nény M, Hantson J, Schubert R, Wiessner C, Quattropani A, Beher D. 2022 O-GlcNAcase inhibitor ASN90 is a multimodal drug candidate for tau and α-synuclein proteinopathies. ACS Chem. Neurosci. **13**, 1296–1314. (10.1021/ACSCHEMNEURO.2C00057/ASSET/IMAGES/LARGE/CN2C00057_0009.JPEG) 35357812 PMC9026285

[99] Marotta NP, Lin YH, Lewis YE, Ambroso MR, Zaro BW, Roth MT, Arnold DB, Langen R, Pratt MR. 2015 O-GlcNAc modification blocks the aggregation and toxicity of the protein α-synuclein associated with Parkinson’s disease. Nat. Chem. **7**, 913–920. (10.1038/nchem.2361) 26492012 PMC4618406

[100] Lewis YE, Galesic A, Levine PM, De Leon CA, Lamiri N, Brennan CK, Pratt MR. 2017 O-GlcNAcylation of α-synuclein at serine 87 reduces aggregation without affecting membrane binding. ACS Chem. Biol. **12**, 1020–1027. (10.1021/acschembio.7b00113) 28195695 PMC5607117

[101] Levine PM, De Leon CA, Galesic A, Balana A, Marotta NP, Lewis YE, Pratt MR. 2017 O-GlcNAc modification inhibits the calpain-mediated cleavage of α-synuclein. Bioorg. Med. Chem. **25**, 4977–4982. (10.1016/j.bmc.2017.04.038) 28487126 PMC5603368

[102] Levine PM, Galesic A, Balana AT, Mahul-Mellier AL, Navarro MX, Leon CA, Lashuel HA, Pratt MR. 2019 α-Synuclein O-GlcNAcylation alters aggregation and toxicity, revealing certain residues as potential inhibitors of Parkinson’s disease. Proc. Natl Acad. Sci. USA **116**, 1511–1519. (10.1073/PNAS.1808845116/SUPPL_FILE/PNAS.1808845116.SAPP.PDF) 30651314 PMC6358670

[103] Balana AT *et al*. 2024 O-GlcNAc forces an α-synuclein amyloid strain with notably diminished seeding and pathology. Nat. Chem. Biol. **20**, 646–655. (10.1038/s41589-024-01551-2) 38347213 PMC11062923

